# A new module in the drug development process: preclinical multi-center randomized controlled trial of R-ketamine on alcohol relapse

**DOI:** 10.1038/s41386-025-02071-w

**Published:** 2025-02-28

**Authors:** Marcus W. Meinhardt, Ivan Skorodumov, Jérôme Jeanblanc, Federica Benvenuti, Fahd François Hilal, Esi Domi, Camille André, Sandra Bodeau, Virginie Jeanblanc, Kevin Domanegg, Roberto Ciccocioppo, Mickael Naassila, Rainer Spanagel

**Affiliations:** 1 Institute of Psychopharmacology, Central Institute of Mental Health, Medical Faculty Mannheim, University of Heidelberg, Heidelberg, Germany; 2 Department of Molecular Neuroimaging, Central Institute of Mental Health, Medical Faculty Mannheim, University of Heidelberg, Heidelberg, Germany; 3 University of Picardy Jules Verne, INSERM UMR1247 research unit, Amiens, France; 4 School of Pharmacy, Center for Neuroscience, Pharmacology Unit, University of Camerino, Camerino, 62032 Italy; 5 Laboratory of Pharmacology and Toxicology, Amiens-Picardie University Hospital, Amiens, France; 6 MP3CV Laboratory, UR UPJV 7517, University of Picardy Jules Verne, Amiens, France; 7 German Center for Mental Health (DZPG), partner site Mannheim/Heidelberg/Ulm, Heidelberg, Germany

**Keywords:** Addiction, Addiction

## Abstract

The drug development process in psychiatry faces significant challenges due to low reproducibility rates in animal testing, which often leads to translation failures. To address this issue, we introduce a new approach in psychiatric drug development: a preclinical randomized controlled trial (preRCT). To demonstrate its potential utility, we conducted a multi-center preRCT using the alcohol deprivation effect (ADE) model to assess the impact of ketamine and R-ketamine on alcohol relapse across three European research centers. Ketamine (20 mg/kg) significantly reduced relapse, while R-ketamine showed efficacy only in females. A higher dose of R-ketamine (40 mg/kg) was also effective in males. These sex-dependent effects were linked to plasma R-ketamine levels, which were two-fold higher in female compared to male rats. Notably, R-ketamine demonstrated a lasting reduction in alcohol consumption without adverse effects. In conclusion, our preRCT demonstrates R-ketamine’s effectiveness in reducing alcohol relapse and supports translation to a clinical RCT that accounts for sex-dependent effects.

## Introduction

The field of biological psychiatry suffers from translation failures as many compounds that have been developed preclinically are often ineffective in clinical trials. This has severely hampered translational research in this field and has had a negative impact on pharmaceutical industry engagement on drug development programs in psychiatry. Several factors can contribute to the limited generalizability of preclinical data, which include animals’ age, experiments often carried out in males only, lack of studies that include chronic drug administration, and other reasons [1]. Recognizing these challenges, researchers advocate for greater attention to data robustness and generalizability in preclinical study design [2, 3].

Recently, we introduced the STRINGENCY framework, which is an integrative approach to good practice guidelines for preclinical alcohol research, which aims to improve preclinical research to better prepare it for translation and minimize the “valley of death” in translational research [4]. One new module of STRINGENCY is the use of multi-site preclinical randomized controlled trials (preRCTs). Our multi-site preRCTs approach follow as much as possible the guidelines of clinical trials and aims for rigorously testing a drug’s clinical potential and restricting the advance of ineffective interventions advanced into clinical testing. Here, we sought to use this framework to conduct a preRCT involving three different European sites; Mannheim (Germany), Camerino (Italy), and Amiens (France). To accomplish this objective, in a three-arm design, we investigated the therapeutic potential of ketamine and R-ketamine in the Alcohol Deprivation Model of alcohol use disorder (AUD).

Ketamine, an N-methyl-D-aspartate receptor antagonist, is a promising candidate therapyfor individuals with AUD. A pilot trial in patients with AUD found that a single ketamine infusion, when combined with behavioral therapy, increased alcohol abstinence, delayed the time to relapse and reduced the amount of heavy drinking days [5]. Similarly, repeated doses of ketamine showed increased abstinence in individuals with severe AUD, although no significant difference in alcohol relapse compared to placebo was found in this study [6]. Ketamine could be also used as a substitution drug by producing ethanol-like effects in detoxified individuals with AUD [7, 8]. Likewise in rats, ketamine and other non-competitive NMDA receptor antagonists such as phencyclidine and MK-801 can substitute for ethanol in a discrimination paradigm [9]. Conversely, alcohol has been demonstrated to trigger a re-emergence of ketamine-like experiences in former ketamine users [10].

Ketamine is a racemic mixture of its two enantiomers R-ketamine (arketamine) and S-ketamine (esketamine), and the latter has recently been approved by the Food and Drug Administration (FDA) and European Medicines Agency (EMA) as a rapid-acting antidepressant drug. Interestingly, preclinical research suggests that R-ketamine may have a more favorable safety profile including a decreased abuse liability, and a more potent and longer-lasting antidepressant effect compared to ketamine and S-ketamine [11–13]. Importantly, R-ketamine has already been used in clinical trials in depression – although the therapeutic effects for this indication were not favorable an excellent safety profile has been reported ([13] & https://atai.life/2023/01/09/atai-life-sciences-announces-results-from-phase-2a-trial-of-pcn-101-S-ketaminee-for-treatment-resistant-depression/). Therefore, R-ketamine can be repurposed for other indication; i.e., for the treatment of AUD.

Here we set out to test the hypothesis that R-ketamine reduces relapse behavior with a good safety profile. A good safety profile for R-ketamine compared to ketamine is of particular importance for patients with AUD because these patients have alterations in the NMDA receptors subunit composition [14–16] and ketamine, which has a greater affinity to NMDARs may potentially cause adverse effects in this molecular constellation.

For doing so, we tested R-ketamine in a well-established rat model of relapse, the alcohol deprivation effect (ADE) model. Following repeated deprivation phases, the ADE is characterized by an increased demand for alcohol intake that results in compulsive drinking behavior [17–21]. The model resembles a relapse situation in patients with AUD and has been validated by the effectiveness of approved anti-relapse compounds such as acamprosate and naltrexone [22–24]. Importantly, rats undergoing the ADE procedure show similar alterations in NMDA receptor subunit composition as those found in patients with AUD [25–28]. Therefore, adverse side effects that may occur in the ADE model following treatment with R-ketamine or ketamine may well be translated to the AUD condition in humans. Here, we applied a multi-center preRCT design that closely follows the guidelines of clinical RCTs to test the effects of ketamine and R-ketamine versus placebo and pre-registered our study design (see study preregistration DOI: 10.17590/asr.0000264).

## Materials and methods

### Design of the multi-site preRCT

For the present study we implemented the novel concept of randomized multi-center preclinical phase II testing in laboratory animals. Trial preregistraition was done ahead of the study (DOI: 10.17590/asr.0000264). We structured the materials and methods section following the SPIRIT recommendations (https://spirit-statement.org/) that are used for clinical studies.

### General study design

Two-month-old male and female Wistar rats were used with a total of 143 female and 154 male rats and the same conditions were applied in Mannheim (Germany), Amiens (France) and Camerino (Italy). For the daily measurements of alcohol consumption all rats were housed individually in standard rat cages under a 12 hour artificial light/dark cycle (lights on at 6:00 a.m.). Room temperature was kept constant (temperature: 22 ± 1 °C, humidity: 55 ± 5%). Standard laboratory rat food and water were provided ad libitum throughout the experimental period. Body weights were measured weekly. All experiments were approved by the institutional Committees on Animal Care and Use, by the Regierungspräsidium Karlsruhe, and were performed in accordance with the European and German, Italian and French national guidelines. To model relapse-like drinking in rats we applied the ADE model as previously published [17, 20]. After two weeks of habituation to the animal room, rats were given *ad libitum* access to tap water and to 5%, 10% and 20% ethanol solutions (v/v) as well. Spillage and evaporation were minimized by the use of special bottle caps. With this procedure the ethanol concentration remains constant for at least one week [29]. The positions of bottles were changed weekly. The first deprivation period was introduced after eight weeks of continuous alcohol availability. After a deprivation period (between 3-4 weeks), rats were given access to alcohol again and two more deprivation periods were introduced in a random manner, i.e., the duration of following drinking and deprivation phases was irregular, i.e. approximately 4 ± 1 week and 2 ± 1 week, respectively in order to prevent adaptive behavioral mechanisms [27, 30]. The long-term voluntary alcohol drinking procedure including all deprivation phases lasted a total of 10 months.

Random allocation of rats to the treatment arms was done separately for each of 6 strata (formed by sex and participating center) using computer-generated lists of permuted blocks (randomization schedule was generated using a custom developed R script resulting in such a way that the mean baseline total alcohol intake was approximately the same in each group). Experimenters had been blinded for the treatment using the EQIPD blinding protocol^1^ and the blinded code was only broken after the analysis of the data has been fully completed.

### Inclusion/ exclusion criteria

The key inclusion criterion was a daily baseline alcohol intake of at least 2.5 g/kg/day to ensure relevant alcohol intake of rats. Exclusion criteria included adverse effects in animal behavior (with the exception of the alcohol-related phenotype) or any other pathological features (e.g. tumors). The GV-SOLAS recommended score sheet^2^ was used to determine exclusion of rats during the study. Other exclusion criteria involved leaky bottles or incorrect study drug application.

### Groups

As preclinical and clinical evidence suggests that sex influences disease trajectories and interventions in AUD patients [31], male and female rats were studied in comparison in our three-arm design: placebo vs. the application of 20 mg/kg R-ketamine and a direct comparator arm with ketamine (20 mg/kg). Preceding the trial, we conducted a dose-response experiment in order to find the optimal dose for the study (Supplementary Fig. 1). As placebo control we used, saline, as ketamine and R-ketamine was diluted in saline solution.

### Interventions

In order to study the effects of ketamine enantiomers in comparison to vehicle and acamprosate as a comparator, rats were divided into groups in such way that the mean baseline total alcohol intake was approximately the same in each group. Baseline drinking was measured daily for one week. After the last day of baseline measurement, the alcohol bottles were removed from the cages, leaving the rats with free access to food and water for the complete deprivation period.

**Fig. 1:**
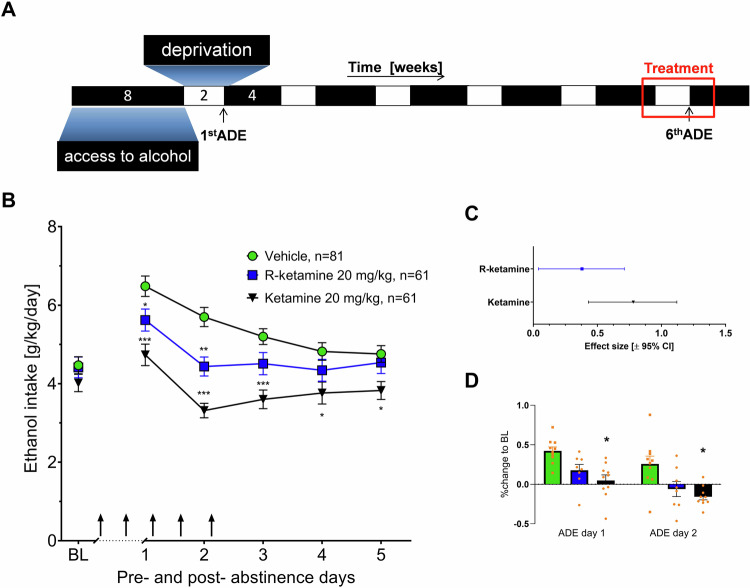
Timeline and results of the preRCT in the whole population (males and females). **A** Numbers indicate weeks of every experimental phase. After habituation first alcohol consumption phase was initiated followed by an alcohol-free period (alcohol deprivation) and re-access (relapse/alcohol deprivation effect) to alcohol. Drug application is highlighted in red. **B** Effects of acute R-ketamine and ketamine on relapse-like drinking. Intake of total ethanol (calculated in g of pure alcohol per kg of body weight per day) before and after a deprivation period of two weeks. The last 3 days measurements of ethanol intake is given as baseline drinking—‘BL’. Arrows indicate the administration of either vehicle, or 20 mg/kg of R-ketamine/Ketamine. In addition effect size for different treatments are displayed in (**C**). **D** Changes in locomotion in percent to baseline are presented indicative for sedative effects of ketamine. All data are presented as means ± SEM. significant differences from the vehicle control group: *P < 0.05, **P < 0.01, ***P < 0.001.

Following the 5th period of abstinence drug testing was performed during the 6th ADE measurement (Fig. 1A). Repeated deprivations have been shown to boost compulsive relapse behavior [17]. For drug testing during the ADE each animal was subjected to a total of five intraperitoneal (i.p.) injections starting at 7 p.m. in the evening before the reintroduction of the alcohol bottles, followed by four applications in 12 h intervals of either vehicle or drug (For a visual depiction of the drug treatment, arrows indicating the injection are shown within Fig. 1B). The alcohol bottles were reintroduced after the second injection (at ~ 9 a.m.) and the occurrence of an ADE was determined. Total ethanol (g/kg of body weight/day) and water intake (ml/kg of body weight/day) were measured daily at ~9 a.m. for the subsequent week. In order to test for persistent treatment effects, both ethanol and water intake were measured for four more weeks in a subset of rats. Each rat’s body weight was recorded 24 h before the first injection and 12 h after the last injection for the calculation of consumption during the relapse phase.

In addition, home cage locomotor activity was monitored by use of an infrared sensor connected to a recording and data storing system (Mouse-E-Motion, Infra-e-motion, Henstedt-Ulzburg, Germany). A Mouse-E-Motion device was placed above each cage (30 cm from the bottom) so that the rat could be detected at any position inside the cage. The device was sampling every second, whether the rat was moving or not. The sensor could detect body movement of the rat of at least 1.5 cm from one sample point to the successive one. The data measured by each Mouse-E-Motion device were downloaded into a personal computer and processed with Microsoft Excel.

### Outcomes

#### Primary

The primary endpoint (efficacy of the treatment with reduction of relapse) was defined as the reduction of ADE in the first 5 days after re-access to alcohol after treatment in both sexes with a low side effect profile.

#### Secondary outcomes

Secondary endpoints were defined as reduction of homecage drinking at four weeks after administration of the therapeutic dose (long-term follow-up endpoints), sex differences in the efficacy of R-ketamine and the determination of plasma metabolite concentrations in males and females.

#### Sample size

A sample size calculation was performed prior to the experiments based on previous data sets on ADE relapse data of various compounds used in the past 15 years (effect size of d = 0.52). With an alpha of 0.05 and power of 0.9 resulted in a sample size of *n* = 58 per group, including both sexes. To ensure that each of the strata defined in terms of center shall be equally represented in all arms of the trial an n of at least 10 rats per site (per arm and sex) was considered.

#### Compounds

Alcohol drinking solutions were prepared from 96% ethanol and then diluted with tap water. The following drugs were used in the study: R/S-ketamine (here always referred to ketamine), S-ketamine (both: Merck, Darmstadt, Germany), and R-ketamine (Atai Life Sciences, Berlin, Germany). All drugs were dissolved in saline (Braun, Melsungen AG).

#### LC-MS/MS method of ketamine plasma levels

A further important feature of clinical studies is the measurement of drug and metabolites plasma levels. In order to study metabolic differences between males and females rats, dosage of plasma concentrations of ketamine and its metabolites were performed by LC-MS/MS. We thus determined plasma levels of ketamine enantiomers (sampling 15 min post application of a single injection of the respective ketamine enantiomer via tail vein) from a separate cohort of satellite rats and its main metabolites (Norketiamine (NK), Hydroxynorketamine (HNK) and Dihydroxynorketamine (DHNK)) by a liquid chromatography tandem mass spectrometry (LC-MS/MS) method (8060 LC-MS/MS, Shimadzu®, Marne-la-Vallée, France). Briefly, 25 µL of rat plasma were diluted 4× in NaCl before the deproteinisation step. Then ketamine and its metabolites were extracted from 50 µL of diluted plasma by 100 µL of an ice-cold acetonitrile solution containing the deuterated internal standard (d4-Ketamine). Samples were stored during ten minutes at -20°C before addition of 20 mg of QuEChERS® salts (QuEChERS Method EN 15662 – Mylar pouch, UCT). After centrifugation, 30 µL of the supernatant was diluted 4-fold in mobile phase before the injection in the chromatographic system (injection volume: 10 µL). Chromatographic separation was performed at 60 °C on an Acquity UPLC BEH C18 1.7 µM (2.1 × 50 mm, Waters®, Saint-Quentin-en-Yvelines, France). The column was eluted with a gradient of mobile phase B (B: isopropanol/acetonitrile [90/10, v/v]) and mobile phase A (A: ultrapure water with 0.02% formic acid and 2 mM ammonium formiate) at a flow rate of 0.3 mL/min. Data were acquired in multiple reaction monitoring (MRM) mode after ionization in negative electrospray ionization mode. MRM transitions used for ketamine, NK, HNK and DHNK quantification were respectively: 238.2–>125.1, 224.2–>125.1, 240.2–>151.1 and 221. 8–>205.1.

#### Statistics

Data derived from home-cage drinking (total alcohol intake and water intake) and home-cage locomotor activity were analyzed using a two-way repeated measures Analysis of Variance (ANOVA), with treatment as the between-subject factor and day/week as the within-subject factor. Data from males and females were pooled and sex was added as covariate. We did not have any missing data points of any animal that went into the analysis. In general, we tested data for skewness using the Shapiro-Wilk test and repeated measures ANOVA were tested for sphericity using Mauchly’s test. When appropriate, corrections in using Greenhouse-Geisser for ε < 0.75 and Huynh-Feldt for ε > 0.75 were performed. To visualize differences between sexes, individual subgroup graphs for each sex are provided in each experiment. Locomotion, alcohol and water intake on post-treatment days were expressed as the percentage relative to baseline drinking. The effect size was calculated as d = (M2 − M1)/SDpooled. Whenever significant differences were found, post-hoc Newman–Keuls tests were performed. All statistical analyses were conducted with Statistica 13.3 (Statsoft, Hamburg, Germany).

## Results

### R-ketamine reduces relapse in the ADE model

Throughout the study, a total of 154 male and 143 female rats were used among three independent European research centers, the Central Institute of Mental Health Mannheim, Germany, University of Amiens, France and the University of Camerino, Italy. None of the rats died during the experiments, and none were excluded from the analysis.

Based on the preceding dose-response experiment comparing 10 vs. 20 mg/kg R-ketamine in a cohort of female rats (Supplementary Fig. 1), we decided to use the 20 mg/kg dose for the preRCT.

The analysis of the combined multi-site study from Mannheim, Amiens, and Camerino revealed that repeated administration of 20 mg/kg of ketamine or 20 mg/kg R-ketamine in the whole study population (males and females) resulted in a reduction in alcohol intake compared to vehicle (Fig. 1B). Alcohol intake was significantly reduced in both groups as confirmed by the significant treatment day × treatment interaction effect [R-ketamine: F(3.01,421.54) = 5.33, *p* < 0.002; ketamine: F(3.05,427.71) = 13.11, *p* < 0.001] compared with the placebo group. However, while ketamine induced a significant decrease in alcohol consumption throughout all 5 days following abstinence (post-hoc Neuman Keuls), the effect of R-ketamine was less pronounced and reached significance only on the first two days post-abstinence. To better compare treatment efficacy for each treatment we calculated the effect sizes for R-ketamine 0.38 [0.04–0.71; 95% CI] and 0.78 [0.43–1.12; 95% CI] for ketamine (Fig. 1C).

To measure ketamine and R-ketamine sedative side effects, all rats were further investigated for locomotion difference between treatment groups. We observed a significant locomotion impairment in rats treated with ketamine [effect of treatment: F(1,18) = 16.259, *p* = 0.001] which was also lower but not significant in rats treated with R-ketamine [treatment effect: F(1, 15) = 3.9452, *p* = 0.065] (Fig. 1D).

### Sex differences in the efficacy of R-ketamine and ketamine on alcohol relapse

**Fig. 2:**
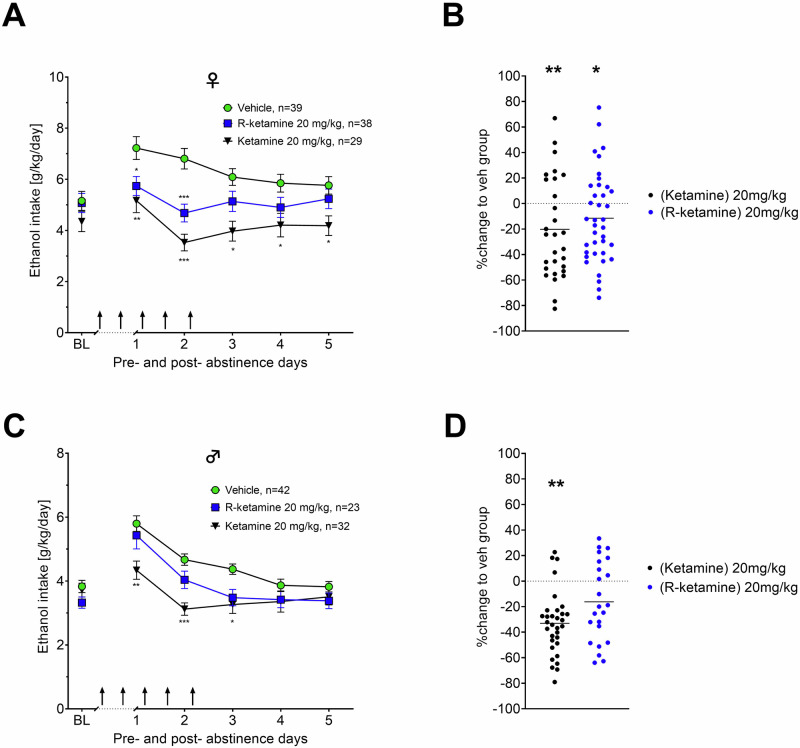
Sex differences of R-ketamine and ketamine on relapse-like drinking within the preRCT. ADE measurement by intake of total ethanol (calculated in g of pure alcohol per kg of body weight per day) for female (**A**, **B**) and male (**C**, **D**) rats respectively. The last 3 days measurements of ethanol intake is given as baseline drinking - ‘BL’. Arrows indicate the administration of either vehicle, or 20 mg/kg of R-ketamine/ketamine. show the efficacy to reduce relapse (in percent to baseline). Data are presented as means ± SEM. significant differences from the vehicle control group: *P < 0.05, **P < 0.01, ***P < 0.001.#.

Considering previous findings showing that ketamine reduces alcohol binge drinking in female mice but not male mice [32], our study further aimed to investigate as a secondary endpoint, whether R-ketamine and ketamine exhibit sex-dependent effects on relapse in our ADE rat model. In female rats, the administration of 20 mg/kg of R-ketamine resulted in a significant reduction of ADE [effect of treatment: F(1,75) = 6.2251, *p* = 0.01479; days x treatment interaction: F(3.19,239.14) = 5.8900, *p* = 0.0005] following abstinence compared to vehicle (Fig. 2A, B). Ketamine had a more pronounced effect than R-ketamine throughout all five days in female rats [effect of treatment: F(1,66) = 17.687, *p* < 0.001; days × treatment interaction: F(2.77,183.21) = 6.3859, *p* < 0.001]. In contrast, in male rats ketamine reduced the ADE only during the first three days following abstinence [effect of treatment: F(1,72) = 12.624, *p* < 0.001; days × treatment interaction: F(3.31,238.21) = 9.0451, *p* < 0.001]. For R-ketamine, a weaker, close to significant effect of treatment was found over all days [F(1,63) = 3.8434, *p* = 0.05437] (Fig. 2C). To better compare treatment efficacy for each treatment in a sex-dependent manner we plotted % change of alcohol consumption to the vehicle group in Fig. 2B (female rats) and 2D (male rats). These findings collectively suggest that both R-ketamine and ketamine are more effective in reducing alcohol consumption in females compared to males. One potential explanation for these sex differences are differences in pharmacokinetics of ketamine and its metabolites in male and female rats.

We did an additional study in both sexes that was not part of the preRCT. In this study, we investigated whether a prophylactic administration of R-ketamine or ketamine (7 days and 1 day before abstinence) is also able to reduce the consumption of alcohol in our ADE rat model. All treated groups showed the expected increase in alcohol consumption, indicating occurrence of an ADE. A two-way ANOVA for repeated measures showed a significant increase in alcohol intake after the deprivation phase in all animal groups as compared to basal drinking [factor day for males: F(6,150) = 26.01, *p* < 0.0001; Females: F(6,144) = 12.98, *p* < 0.0001] (Supplementary Fig. S2). However, there was no difference of either R-ketamine or ketamine treatment to the vehicle group in male and female rats on ADE. Measurements on potential side effects of the treatment, (total fluid intake and locomotion) also showed no differences between groups (data not shown).

### Sex-dependent differences in the pharmacokinetics of ketamine enantiomers and its metabolites

**Fig. 3:**
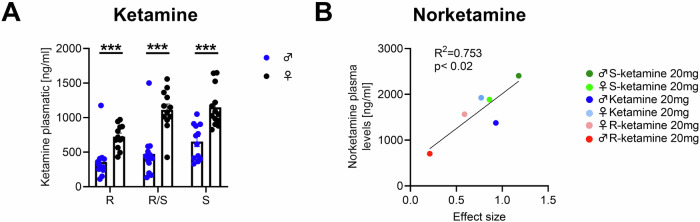
Pharmacokinetic analysis of different ketamine enantiomers in males and females. Plasma concentrations of ketamine, R-ketamine and S-ketamine (**A**) and their main metabolite norketamine (**B**) in male and female rats measured 15 min after intra-peritoneal administration of the respective enantiomers. Data are presented as means ± SEM, ***P < 0.001.

Male and female rats display differences in the pharmacokinetics of ketamine. Specifically, female rats exhibited longer half-lives and slower clearance rates of ketamine, leading to higher concentrations of ketamine and its major active metabolite, norketamine, in both the brain and plasma when compared to male rats [33]. Based on our sex-dependent findings of R-ketamine, we explored potential sex-dependent differences in plasma concentration. We investigated sex-dependent pharmacokinetic profiles of both ketamine enantiomers as well as their metabolites. Our findings demonstrate that plasma levels of ketamine enantiomers were higher in female rats compared to males [R-ketamine: F(1,23) = 16.730, *p* = 0.00045; ketamine: F(1,23) = 24.736, *p* = 0.00005; S-ketamine: F(1,23) = 21.040, *p* = 0.00013; Fig. 3A]. Moreover, the major active metabolite of ketamine, norketamine showed considerable sex-dependent differences. Thus, the different ketamine enantiomers show a significant correlation to the effect size in reducing relapse in a sex-dependent manner (F(1,4) = 12.20, *p* = 0.0251; Fig. 3B). A complete dataset of all metabolites, including hydroxynorketamine as well as dihydroxynorketamine can be found in supplementary table 1. Considering these differences in metabolites and that female rats exhibited plasma concentrations more than two-fold higher than those of males (Fig. 3A), we decided to investigate a further male cohort with a concentration of 40 mg/kg R-ketamine.

### Efficacy of 40 mg/kg R-ketamine in male rats

**Fig. 4:**
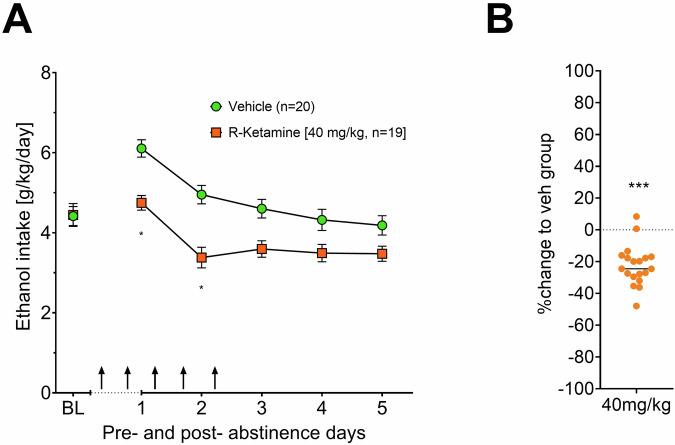
Effects of acute R-ketamine (40 mg/kg) on relapse-like drinking in male rats. Intake of total ethanol (calculated in g of pure alcohol per kg of body weight per day) (**A**) before and after a deprivation period of two weeks. The last 3 days measurements of ethanol intake is given as baseline drinking - ‘BL’. Arrows indicate the administration of either vehicle or 40 mg/kg of R-ketamine. **B** Shows the efficacy to reduce relapse (in percent to baseline). Data are presented as means ± SEM. significant differences are display in relation to the vehicle control group, *P < 0.05, **P < 0.01, ***P < 0.001.

In order to study the potential acceleration of ketamine metabolism or clearance in male rats, we decided to investigate efficacy and safety of R-ketamine on ADE in a further male cohort treated with an increased dose of 40 mg/kg R-ketamine. This dose testing was not part of the pre-RCT study. The results have been derived from the two study sites in Mannheim and Camerino. Indeed 40 mg/kg of R-ketamine significantly reduced alcohol consumption in male rats [effect of treatment: F(1,37) = 17.015, *p* < 0.001; days × treatment interaction: F(4, 148) = 3.2889, *p* = 0.01291; Fig. 4A, B] without changing locomotion (Supplementary Fig. S3). These results show that an acute and sex-optimized dose of R-ketamine can reduce relapse-like drinking behavior.

### Efficacy summary of all treatment modalities and long-lasting suppression of alcohol intake by R-ketamine

**Fig. 5:**
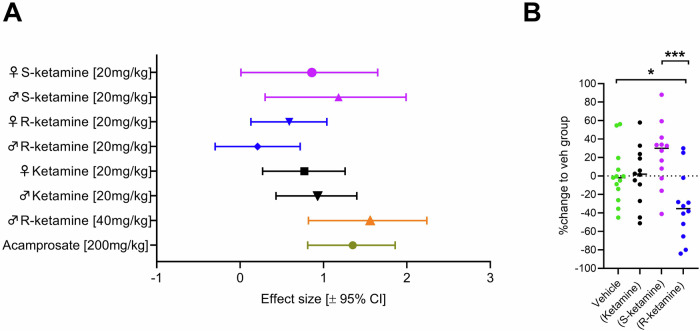
Summary of efficacy data and potential long-lasting effect of drug treatment. **A** Drug treatment of all treatment modalities displayed for their first day of alcohol relapse represented in effect size to responding of the respective vehicle-treated group. **B** Long-term effects of treatment with different ketamine enantiomers on baseline alcohol intake represented in percentage change to drinking behavior of the respective vehicle-treated group. Color coding represents: Green = Saline, purple = S-ketamine [20 mg/kg], blue = R-ketamine [20 mg/kg], black = ketamine [20 mg/kg], orange = R-ketamine [40 mg/kg]. Data are presented as means, significant differences from the vehicle control group: *P < 0.05.

To summarize the data generated in this multi-site preRCT, we compared the results obtained in terms of their effectiveness in reducing alcohol relapse on the first day of alcohol re-access. In addition to the aforementioned studies, we further examined rats in a separate cohort that received the S-enantiomer to create a comprehensive dataset. All ketamine enantiomers demonstrated efficacy in reducing alcohol relapse [ketamine: F(1,140) = 20.877, *p* < 0.001; S-ketamine: F(1,48) = 9.9716, *p* = 0.00275; R-ketamine 20 mg/kg: F(1,140) = 4.9664, *p* = 0.02744; R-ketamine 40 mg/kg: F(1,37) = 22.556, *p* < 0.001]. Ketamine exhibited the highest reduction in alcohol relapse (−26.9%), followed by S-ketamine (−22.6%), R-ketamine 40 mg/kg (−22.3%), and R-ketamine 20 mg/kg (−13.3%). In addition, we summarized the effect size of all ketamine enantiomers (Fig. 5A) where the 40 mg/kg dose of R-ketamine (male rats) had the numerically largest effect size of 1.56 [0.82–2.24; 95% CI]. We then did a comparison to acamprosate which is a clinically used anti-relapse medication [34] and which has frequently been tested in our ADE model [23, 24]. For this purpose, we used our ADE database and calculated for 86 rats of previously conducted and published studies that were treated with a standard dose of 200 mg/kg acamprosate a reduction in alcohol relapse by −28% and calculated an effect size of 1.35± (95% CI 0.5) for acamprosate for male rats in the ADE model (Fig. 5A).

We also summarized and compared data obtained between different study sites for center effects (Supplementary table 2). Ketamine had a very similar effect size across all sites (Mannheim (d = 0.99), Amiens (d = 0.97) and Camerino (d = 1.07)). R-ketamine results for the 20 mg/kg dose varied more between sites (Mannheim (d = 0.77), Amiens (d = 0.41) and Camerino (d = 1.12)). Finally, the higher 40 mg/kg R-ketamine dose was only tested in Camerino and Mannheim and again resulted in very similar effect sizes (Mannheim (d = 1.55) and Camerino (d = 1.67)).

Finally, we investigated persistent drug effects on alcohol consumption in all treatment groups as secondary outcome at the site in Amiens. Despite ketamine demonstrating the highest efficacy in reducing alcohol relapse, baseline drinking 4 weeks after treatment did not differ from that of vehicle-treated rats (F(1,23) = 0.00182, *p* = 0.966; Fig. 5B). Similarly, S-ketamine showed no long-term effects on alcohol consumption (F(1,23) = 3.3439, *p* = 0.081). In contrast, R-ketamine suppressed alcohol intake in the 4 week follow-up period, indicating long-lasting efficacy (F(1,23) = 6.1487, *p* = 0.021) (Fig. 5B).

## Discussion

There is a great need for novel drug discovery in psychiatry. Usually, preclinical animal studies are used to define new drug targets and to test new molecules that engage on those targets. However, translating animal studies into the clinics can often lead to translation failures. The large gap between basic scientific research and translation to novel therapeutics has been described as the “valley of death” [35]. To overcome these challenges we here present a novel module in the drug development process, namely a preclinical randomized controlled trial (preRCT). The core concept of this new module is that aligning the study design of a clinical trial with the design of a preclinical animal study holds the potential for minimizing translation failures. Thus, our preRCT design closely mirrors the design of a clinical trial using the SPIRIT guidelines including preregistration, power analysis resulting in sufficient number of rats to retrieve meaningful effect sizes, multi-site testing, full-blinding, male-female comparison, and plasma level monitoring of the drug of interest and its metabolites. Our preRCT approach also follows the FAIR Guiding Principles [36]. FAIR improves the Findability, Accessibility, Interoperability, and Reuse of digital datasets (www.go-fair.org). Initially FAIR focused on datasets from human research and trials; very recently the guidelines were also adapted to preclinical research [37]. In summary, the design of our preRCT resembles that of a clinical trial making it as a valuable new module in the drug development process to better translate preclinical animal testing to human applications.

To illustrate the power of our new preRCT module, we conducted a three-arm, multi-center study to investigate the effects of R-ketamine versus ketamine and saline control on relapse-like alcohol drinking behavior (here measured by ADE) in female and male rats. The results in our multi-site preRCT in 297 rats revealed that administering repeated injections of 20 mg/kg ketamine and R-ketamine led to a significant reduction in alcohol consumption during an ADE. However, the study dose of 20 mg/kg R-ketamine exhibited a smaller reduction in alcohol intake in male compared to female rats. Preclinical animal studies consistently demonstrate that females respond to lower doses of ketamine compared to males [38, 39]. We hypothesized that female and male rats might have distinct pharmacokinetic profiles for ketamine that would explain these sex differences. Indeed, plasma concentrations of ketamine and its metabolites revealed large sex differences that were highly correlated with the effect sizes. Especially, given that ketamine’s metabolites, such as norketamine - which is a behavioral active metabolite - are believed to play a pivotal role in the therapeutic mechanism via targeting NMDARs [40], the observed higher levels in females could account for the enhanced efficacy of R-ketamine in reducing relapse. Importantly, in a follow-up experiment in which we doubled the R-ketamine dose to 40 mg/kg, we were able to show a significant relapse prevention effect also in male rats.

While our study suggests that R-ketamine’s effect may be mediated through NMDARs, it is important to contextualize this within prior studies on memantine and its analog neramexane in individuals with AUD. A previous RCT with the NMDA antagonist neramexane conducted across 19 specialized centers with 236 randomized patients revealed no significant benefit of neramexane over placebo in promoting continuous abstinence after 12 weeks of treatment [41]. However, the lack of efficacy may have been attributable to subtherapeutic dosing, as higher doses may be necessary in substitution therapy paradigms. Furthermore, alterations in NMDA receptor subunit composition in alcohol-dependent individuals, such as increased NR1/NR3A subunits that exhibit reduced sensitivity to channel blockers, could have limited the drug’s effectiveness [42]. Notably, post-hoc analyses indicated that patients with higher neramexane plasma levels achieved better abstinence rates, reinforcing the need for adequate dosing. This rationale extends to memantine, suggesting that higher doses may be more effective when used as substitution therapy [43]. However, although higher doses of memantine (>40 mg) have previously been shown to reduce alcohol craving, they increased alcohol consumption for some cases [44]. These insights highlight the complexities of targeting NMDARs in alcohol dependence and underscore the necessity of careful dose optimization in future studies.

The clinical use of ketamine is limited due to its side effects, which include dissociative effects, bladder damage and abuse potential [45]. In rodents, R-ketamine produces less side-effects and has no drug abuse liability compared to ketamine and S-ketamine [11, 46, 47]. In accordance to these findings, rats treated with R-ketamine in our preRCT and subsequent further experiments did not display impairments in locomotion whereas ketamine produced side-effects. Particularly noteworthy is the finding that R-ketamine, but not ketamine, reduced alcohol consumption even 4 weeks after treatment, demonstrating its long-lasting effectiveness.

The long-lasting effects of R-ketamine that were observed in a small follow-up study may not be mediated by a NMDAR mechanism it is rather better explained by long-lasing metabolites. Especially, dihydroxynorketamine (DHNK) can be a potential candidate as it exerts mGlu2 receptor-dependent effects [48] and mGluR2 plays a critical role in relapse behavior [49–52].

A very much overlooked phenomenon in preclinical studies is the occurrence of a placebo effect in clinical trials that can have a considerable effect size in different psychiatric conditions [53] including AUD [54]. Thus, in a recent meta-regression analysis in 2000 individuals with AUD the placebo response in abstinence rate varied from 17% to 37% in mild to-moderate and high severity patients, respectively. These pronounced placebo effects are not considered in preclinical trials since a placebo effect cannot occur in laboratory animals unless they are first conditioned to the drug [55, 56]. Therefore, it can be assumed that the absence of placebo effect in preclinical intervention studies results in a considerable overestimation of the effect size in the translation in human clinical trials. This suggests a significant overestimation of effect sizes in our preRCT by 17–37%, due to the absence of a placebo response and lower heterogeneity in rats that should be considered for the design of clinical studies.

We recognize the fact that our preRCT has several limitations: (i) As discussed, the absence of a placebo effect in animal experiments represents a limitation that can, however, be overcome by the introduction of a correction factor.(ii) The large genetic and epigenetic heterogeneity seen in a human study population is not well captured by a specific outbred rat strain. One way to overcome this challenge is the use of the heterogeneous stock (HS) rats in future preRCTs. HS rats are highly recombinant animals, established by crossbreeding eight genetically diverse founder strains [57], resulting in a diversity that better mimics the diversity found in a human study population. (iii) Multi-site preRCTs are cost-intensive and require a well harmonized collaborative effort. Moreover, there are only very limited funding schemes that would support such an approach. Nevertheless, we have now build a national and European network of 6 centers for future drug testing. (iv) In our preRCT study design we did not include S-ketamine (only in a secondary study) which would have resulted in a 4-arm design making it more unlikely to be repeated in a clinical trial. (v) We did not run a clinically relevant comparator such as acamprosate. However, we included a large dataset of acamprosate treated rats form previous studies for a better interpretation of the data. (vi) This publication includes additional studies that were not preregistered or part of the pre-RCT, such as the 40 mg/kg R-ketamine experiment in male rats and a cohort of rats that were treated with S-ketamine. (vii) Finally, the 40 mg/kg dose of R-ketamine was only used in male- but not female rats, which also limits the interpretation of the study results.

In summary, we introduce the preRCT as a module in the drug development process for novel psychiatric medication and illustrate the use of a preRCT by testing enantiomers of ketamine in an animal model of alcohol relapse. We conclude that R-ketamine may be a promising and safe drug for relapse prevention in individuals with AUD taking into account gender differences. Future clinical testing is now warranted.

## Supplementary information

SUPPLEMENTAL figures and tables

Preregistration

## Data Availability

Raw data can be found at 10.5281/zenodo.13939056.

## Footnotes

1 https://paasp.sharepoint.com/:w:/s/EQIPD/EZbRvZmZGoRGtsjc1Wk_XOsBLsnNAbg-FBFVj9h199oYMA?rtime=0Pyue-3N3Eg

2 https://www.gv-solas.de/wp-content/uploads/2020/02/Moeglichkeiten-der-Belastungsbeurteilung-im-Tierversuch_2020.pdf
