# Supplementary material for: A new module in the drug development process: preclinical multi-center randomized controlled trial of R-ketamine on alcohol relapse
Source: Neuropsychopharmacology. 2025 Feb 28;50(6):886–94. doi: 10.1038/s41386-025-02071-w (PMC12032358; doi:10.1038/s41386-025-02071-w)
Supplement: Supplementary file 1 — SUPPLEMENTAL figures and tables [file 41386_2025_2071_MOESM1_ESM.pdf]

Supplementary Materials for

A New Module in the Drug Development Process: Preclinical Multi-center Randomized Controlled Trial of R-ketamine on Alcohol Relapse

Marcus W Meinhardt et al.

*Corresponding author. Email: rainer.spanagel@zi-mannheim.de

This PDF file includes:

Figs. S1 to S3
Tables S1 to S2

Fig. S1.

Supplementary Figure 1: Effects of acute R-ketamine 10 vs. 20 mg/kg on relapse-like drinking. Intake of total ethanol calculated in g of pure alcohol per kg of body weight per day) is shown. The average of the last 3 days measurements of ethanol intake is given as baseline drinking—'BL'. BL is followed by a two-week deprivation period. The 5 days following deprivation show the occurrence of an ADE. Arrows indicate the administration of either vehicle, 10 or 20 mg/kg of R-ketamine. Data are presented as means ±SEM. Significant differences from the vehicle control group:*= $P<0.05$.

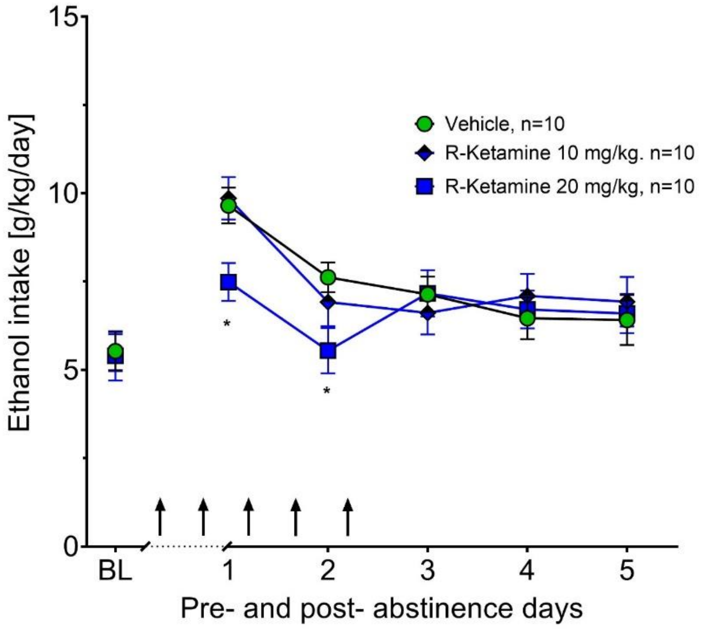

Fig. S2.

Figure S2: **The prophylactic effects of 20 mg/kg R-ketamine and ketamine on alcohol relapse**. Intake of total ethanol (calculated in g of pure alcohol per kg of body weight per day) for each sex before and after a deprivation period of 4 weeks. The last 3 days measurements of ethanol intake is given as baseline drinking - ‘BL’. Arrows indicate the administration of either vehicle, R-ketamine [20mg/kg] or ketamine [20mg/kg] (n=9 per treatment condition and sex). Data are presented as means±SEM.

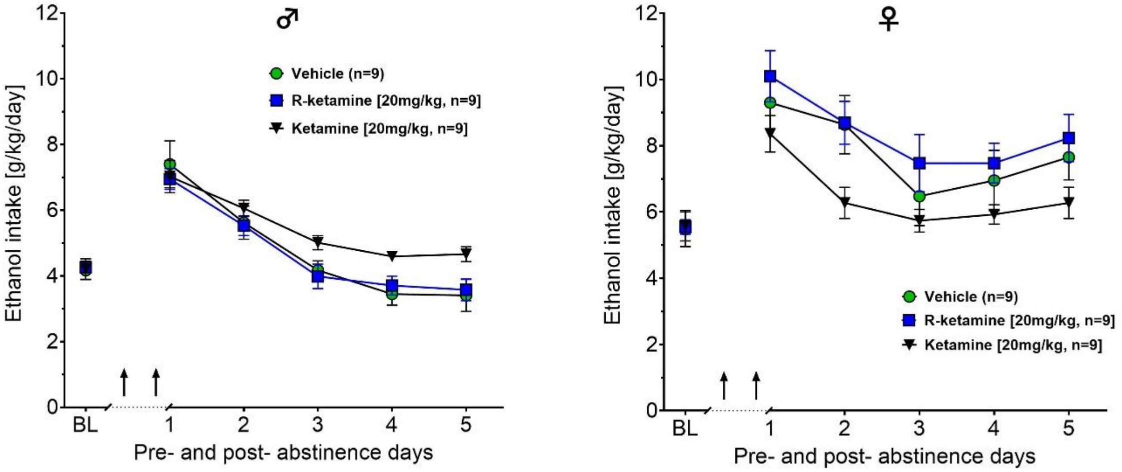

Figure S3: **Effects of acute R-ketamine (40 mg/kg) on locomotion in male rats.** Locomotion in percent change to baseline locomotion after a deprivation period of two weeks. The last 3 days measurements of ethanol intake before deprivation is given as baseline drinking. The change in locomotion is represented for the first and second day of relapse (ADE) for both vehicle (green bars) and R-ketamine (40mg/kg, orange bars). Data are presented as means ±SEM.

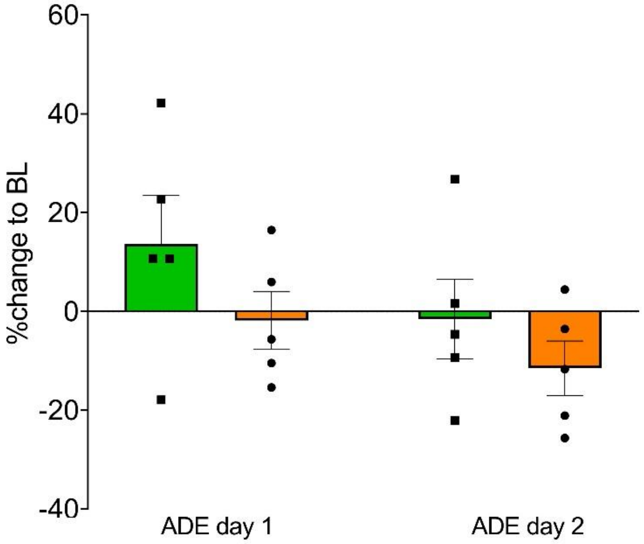

Table S1.

Table S1: Summary of pharmacokinetic data of male and female rats. Data are shown in ng/mg for every metabolite. NorK = Norketamine; HNK = Hydroxynorketamine; DHNK = Dihydroxynorketamine.

Drug	Sex	Metabolite [ng/mg]			
		Ketamine	NorK	HNK	DHNK
Ketamine	Female	1112,59	1925,50	932,48	5,74
Ketamine	Male	473,78	1375,63	1395,31	71,88
R-ketamine	Female	725,66	1565,64	618,21	7,29
R-ketamine	Male	358,87	703,73	638,79	91,81
S-ketamine	Female	1148,33	1885,27	498,99	5,41
S-ketamine	Male	652,27	2405,72	1609,79	60,67

Table S2.

Table S2: Summary of efficacy data of every centre involved in the trial.

Site	Drug	Dose	Treatment group [alcohol intake [g/kg]		Control group [alcohol intake [g/kg]		Effect Size	Confidence Interval for Effect Size	
		[mg/kg]	mean	SD	mean	SD		lower	upper
Mannheim	Ketamine	20	6,83	1,81	8,58	1,73	0,99	0,29	1,64
Amiens	Ketamine	20	3,16	1,07	4,34	1,33	0,97	0,38	1,54
Camerino	Ketamine	20	4,63	1,99	6,66	1,84	1,07	-0,07	2,08
Mannheim	R-ketamine	20	7,38	1,39	8,58	1,73	0,77	0,10	1,42
Amiens	R-ketamine	20	3,82	1,23	4,34	1,33	0,41	-0,15	0,95
Camerino	R-ketamine	20	4,94	1,10	6,66	1,84	1,12	-0,03	2,13
Mannheim	R-ketamine	40	4,95	0,73	6,25	0,94	1,55	0,49	2,47
Camerino	R-ketamine	40	4,52	0,77	5,96	0,93	1,67	0,56	2,63
