# Supplementary material for: A new module in the drug development process: preclinical multi-center randomized controlled trial of R-ketamine on alcohol relapse
Source: Neuropsychopharmacology. 2025 Feb 28;50(6):886–94. doi: 10.1038/s41386-025-02071-w (PMC12032358; doi:10.1038/s41386-025-02071-w)
Supplement: Supplementary file 2 — Preregistration [file 41386_2025_2071_MOESM2_ESM.pdf]

Preclinical Phase II Assessment of R-Ketamine in Alcohol Addiction

Dr Marcus Meinhardt
Central Institute of Mental Health, Quadrat J 5, 68159 Mannheim, Germany

Summary

Worldwide two billion people drink alcohol regularly. A major health consequence is **alcohol addiction and alcohol use disorder (AUD)** that is characterized by chronic relapses. Preventing relapse is the main treatment goal. Current pharmacological treatments have limited efficacy, thus better treatments and prediction approaches that can be easily translated into the clinical situation are warranted. Due to positive results of first clinical and preclinical trials with psychedelic drugs as treatment for various psychiatric disorder, great hope lies in the potential of psychedelics to effectively treat AUD.

Here we propose a **multi-site placebo-controlled trial in rats** as a preclinical confirmatory proof. We aim to confirm the hypothesis that treatment with R-Ketamine will have long-lasting positive effects on relapse behaviour in animal models of alcohol addiction.

Registration details	
Status of the study	Registered
Date of registration	2021-08-03
Date of publication	Not provided
DOI	10.17590/asr.0000264
Planned start of the study	2021-07-21
Planned end of the study	2022-07-20
License	All rights reserved.

1. General Information

Keywords

Alcohol addiction, relaspe, Alcohol deprivation effect, treatment, R-Ketamine

Funding sources

BMBF funded ERA-NET program: Psi-Alc

DOI 10.17590/asr.0000264

International code of classification

6C40.2

2. Study design

Introduction

Alcohol use and abuse account for a tremendous burden of disease worldwide. Excessive alcohol drinking is a leading risk factor for chronic non-communicable diseases and is in fact linked to more than 60 diseases (Rehm et al., 2009). As a consequence of excessive alcohol use 76 million adults worldwide are alcohol dependent (Rehm et al., 2009). Alcohol addiction is characterized by cycles of excessive alcohol consumption, interspersed with intervals of abstinence, and frequent relapses (Heilig et al., 2010). Relapse is a key feature of this disease and relapse prevention is therefore the main objective in the treatment of alcohol dependence. In many countries, naltrexone, nalmefene and acamprosate are approved anti-relapse medications to treat alcohol addiction. However, these drugs have small to medium effect sizes and only a minority of patients benefit from these treatments (van Amsterdam and van den Brink, 2013; Fertig et al., 2018). Thus, easily translatable new drugs into clinical care are warranted.

Due to positive results of first clinical and preclinical trials for different psychiatric conditions, currentlygreat hope lies in the potential of psychedelics to effectively treat alcohol use disorder (AUD). Here we propose to test the efficacy of **R-Ketamine** for treating alcohol addiction. Fifty years ago there was extensive research on hallucinogens in the treatment of addiction. However, due to the misuse of hallucinogens during the flower power movement these substances could not further be developed as medicinal products. The past decade has seen a renaissance of research in potential clinical applications of the classic hallucinogen psilocybin and a recent proof-of-concept study for psilocybin in ten alcohol dependent patients provides a rationale for controlled trials with larger samples to investigate efficacy and the underlying mode of action (Bogenschütz et al., 2015). Before going into time-intensive discussions with regulatory bodies, dose finding and finally cost-intensive phase II testing for other substances such as R-Ketamine in alcoholics we propose here a preclinical placebo controlled trial in the here described DSM based rat model with a step-wise translation into alcoholic patients.

Type of research

Confirmatory

Hypothesis of your study

Treatment with R-Ketamine will have a long-lasting positive effect on relapse behavior in an animal model of alcohol addiction

Study design

Intervention

In the DSM ADE model, renewed access to alcohol solutions after a period of deprivation leads to a pronounced (although temporary) increase in voluntary alcohol intake (i.e. the ADE) in animals. In the current study we will perform three experiments with different drug administration schemes before reintroducing the alcohol bottles and assessment of the ADE. The chosen drug administration schemes were as follows:

DOI 10.17590/asr.0000264

Experiment 1: a repeated dosing scheme (i.e. five doses across three days, starting Monday night, followed by administration twice daily on Tuesday and Wednesday) based on an established paradigm used in our previous ADE studies to test alcohol relapse behavior.

Experimental group: 20 mg/kg R-Ketamine, 5 i.p. injections over 3 days

Comparator group: 20 mg/kg R/S-Ketamine, 5 i.p. injections over 3 days

Control group: Saline (2 ml/kg), 5 i.p. injections over 3 days

Primary efficacy endpoint: Reduction of ADE in the first 5 days after re-access to alcohol after treatment.

Key secondary endpoints: (i) Homecage drinking between groups in reference to baseline up to four weeks after re-access to alcohol

Method of blinding

Experimenters will be blinded for the treatment and the blinded code will only be broken after the analysis of the data has been fully completed. The entire preclinical trial design will follow the guidelines on the development of medicinal products for the treatment of alcohol addiction provided by the European Medicines Agency (EMA), will adhere to the standards proposed for confirmatory biomedical research (Dirnagl, 2016), and the publication of results will follow the ARRIVE guidelines.

Method of randomization

Random allocation of animals to the treatment arms will be done using computer-generated lists of permuted blocks (randomization schedule will be generated using a custom developed R script and will be saved as part of the study design). Randomization will occur separately for male and female rats; treatment groups will be matched in respect to baseline drinking.

3. Methods

3.1. The alcohol deprivation effect

Description of the method

Modelling the entire spectrum of a human mental disorder such as addiction in animals is not possible because of its complexity. Instead, we can translate the current psychiatric diagnostic classification systems DSM-5 into objectively and behaviorally measurable parameters and can thereby model key criteria of the disorder. Regarding relapse behavior, this is a straightforward endeavor, as a relapse is defined as the recurrence of a past condition, namely excessive and uncontrolled drug intake after a phase of abstinence. The alcohol deprivation model provides excellent face validity to relapse behavior seen in alcoholics as it resembles a voluntary relapse-like drinking situation referred to as the alcohol deprivation effect (ADE) (Spanagel, 2017). The ADE is measured as the re-presentation of alcohol to the animals after a certain period of deprivation. To induce DSM-5-like behaviors such as compulsiveness during a relapse situation, wanting (craving), impaired behavioral control (loss of control), reduced sensitivity to alternative rewards (motivational deficits), rats must have free access to alcohol for many months and must undergo several deprivation phases (Vengeliene et al., 2014).

We will use a homecage free choice 4-bottle paradigm (with water, 5, 10 and 20% ethanol solutions). All rats will have access to ethanol for 8 weeks followed by two weeks of deprivation and then again

DOI 10.17590/asr.0000264

4 weeks of alcohol access followed by another 2weeks of abstinence etc. By the 6 th ADE rats show compulsive relapse behavior and drug testing starts. Possible long-lasting effects will be studied during the 7 th and 8 th ADE (at both time points no drug intervention will be made).

Narcotic/analgesic treatment

Not applicable

Drugs/substances

R-Ketamine (Atai Life Science, Germany)

Antibodies

Not applicable

Cell lines, viruses, DNA or RNA constructs and bacteria

Not applicable

4. Statistics

4.1. ANCOVA

Assigned method(s)

The alcohol deprivation effect

Main endpoints

ADE on the first 5 days after re-access to alcohol

Secondary endpoints

Not specified

Sample size calculation

A sample size calculation was performed prior to the experiments based on previous data sets from the lab. With an Alpha of 0.05, power of 0.9 and an effect size of d=0.52 resulting in an sample size of n=29 per group is sufficient. To ensure that each of the strata defined in terms of center and sex shall be equally represented in all arms of the trial an n of 10 per site (per arm) is proposed.

Primary statistical analysis

Data derived from home-cage drinking (total alcohol intake and water intake) and home-cage locomotor activity was analyzed using a two-way ANOVA with repeated measures, with treatment as the between-subject (BS) factor and day/week as the within-subject (WS) factor and sex added as covariate. Since male and female rats differ significantly in their drinking behavior, baseline alcohol and water intake outcome variables as well as locomotion were centered to the respective group mean. Locomotion, alcohol and water intake on post-treatment days were expressed as the percentage relative to baseline drinking. Whenever significant differences were found, post-hoc Student Newman Keuls tests were performed. All statistical analyses were conducted with Statistica 13.3 (Statsoft, Hamburg, Germany).

DOI 10.17590/asr.0000264

Exclusion criteria

We will ensure that the study is as naturalistic as possible and that the results of the trial are transferable to general clinical practice. Only healthy animals will be used in our studies and regularly observed for changing phenotypes. The GV-SOLAS recommended sore sheet will be used to determine exclusions of animals during the study. Other exclusion criteria involve leaky bottles or incorrect intranasal administration.

5. Animals

5.1. Rats (Rattus norvegicus)

Animal strain/breed

CIMH wistar DSM5-based rat model of alcohol addiction

Genetically modified

No

Sex

Female
Male

Further characteristics of the animals (e.g. age, body weight, size)

The rat line we will use is the DSM-5 based Wistar rat model of alcohol addiction which will be used to strengthen preclinical power by including additional clinically relevant behaviors such as compulsivity. This animal model has high face and predictive validity (Spanagel, 2017) and is in accordance to (DSM-5). Most importantly, we have already tested many drug targets and more than 50 different putative anti-relapse compounds in this model (Spanagel, 2009). Medications that reduce relapse rates in alcoholics, such as naltrexone, acamprosate, and nalmefene also reduce compulsive drinking during a relapse situation, demonstrating the predictive power of this animal model (Spanagel, 2009; Foo et al., 2019).

Housing conditions

We will use original Makrolon cages type III, dimensions mm: top (outside) 420x260, bottom (inside) 390x230, height: 150mm, floor area: about 900cm ^{2}

Feeders at the cover and behind them a 5cm raised area.

Bottles hold 250ml with ball nipples.

Dark/light cycle (lights on 6am/off/6pm, light intensity 50 lux)
Humidity and room temperature (~50 % and ~22 #,)
Single housing for 12months
Bedding material (ssniff, Espen 2-3mm)
No Environmental enrichment
Cage changing once per week
Feeding practices (Lasvendi, Rodent 16, autoclaved)
Water quality: tap water

DOI 10.17590/asr.0000264

Refinement

Not specified
